# Supplementary material for: Early high-sensitivity troponin elevation in predicting short-term mortality in sepsis: A protocol for a systematic review with meta-analysis
Source: PLoS One. 2024 Oct 25;19(10):e0301948. doi: 10.1371/journal.pone.0301948 (PMC11508495; doi:10.1371/journal.pone.0301948)
Supplement: Attachment — Submitted filename: Response to Reviewers (1).docx [file pone.0301948.s002.docx]

Santiago de Chile, June 25, 2024

**Dr. Sonu Bhaskar**

**Academic editor**

**PLOS ONE**

*Dear Dr. Bhaskar:*

This letter is to submit a new version of the manuscript entitled “*Early High-sensitivity troponin elevation in predicting short-term mortality in sepsis: Protocol for a Systematic Review with Meta-analysis*”, based on the reviewers’ comments and suggestions. The authors express their gratitude to the reviewers since they have contributed to improving the quality of our manuscript. All authors have discussed and carefully taken into consideration each point raised by the reviewers, which are addressed hereby point-by-point:

**Reviewer #1:**

- Comment 1: “*The authors present a systematic review protocol to assess the association of early high-sensitivity serum troponin levels with short-term mortality in patients admitted to ICU because of sepsis with acute organ dysfunction. Some corrections must be made to the manuscript. The inclusion of only studies published in English in an era of online translators and artificial intelligence is not justified in a systematic review, since texts in other languages can be translated into English and the authors' native language with good quality. The correct thing to do is not to restrict it by publication language*.*”*
  - Response: We agree with the reviewer and have decided to delete this criterion from the protocol. Therefore, English restriction was suppressed from the text and the criteria table. (Line 94, Table 3).
- Comment 2: Authors should consider in the methodology *"The evidence for small-study effects is usually considered on a funnel plot, which shows the study estimates (x axis) against their precision (y axis). A funnel plot is usually recommended if there are 10 or more studies", following the reference* [*https://doi.org/10.1136/bmj.k4597*](https://doi.org/10.1136/bmj.k4597)*”*
  - Response: We agree with the reviewer. Hence, we have rephrased the respective segment for better understanding and included the reference besides the already cited ones (Lines 253-259).
- Comment 3: *“Finally, the authors should try to anticipate in a paragraph possible limitations that could compromise the results of the future systematic review, such as observational studies with small samples, with great heterogeneity and without strict criteria for analyzing troponin levels and diagnosing sepsis”*.
  - Response: Thank you for your comment! A new paragraph has been added under the *Discussion* section, which addresses the possible limitations judged more critical by the authors for the work ahead (Line 268-275).

**Reviewer #2:**

- Comment 1: “*4. Have the authors described where all data underlying the findings will be made available when the study is complete?*

*The PLOS Data policy requires authors to make all data underlying the findings described in their manuscript fully available without restriction, with rare exception, at the time of publication. The data should be provided as part of the manuscript or its supporting information, or deposited to a public repository. For example, in addition to summary statistics, the data points behind means, medians and variance measures should be available. If there are restrictions on publicly sharing data—e.g. participant privacy or use of data from a third party—those must be specified.*

*Reviewer #1: Yes*

*Reviewer #2: No*

*Reviewer #3: Yes*

*Reviewer #4: Yes”*

- Response: The authors thank you for the opportunity to explain further this point. In order to better report all the data that will be available for readers, we have modified the statement given in the statistical analysis segment, whereby now it is explicitly stated that not only formulas, raw data, and calculations are to be presented in an online supplement, but code and model specifications as well (Line 218-219)
- Comment 2: *“Line 117: Studies will be first analyzed based on their title and abstract. Papers that pass this primary scrutiny, or whenever it seems insufficient, will be subject to full text assessment by the same reviewers for final selection. (please rephrase how are you going to analyze)”*
  - *Response:* Thanks for your suggestion. To keep the message direct and understandable, we have rephrased the paragraph (Line 117- 122)
- Comment 3: *“Line 120: Basically, interventional and observational primary studies will be deemed eligible if they reported some form of association between troponin levels and mortality in a general clinical context (how are you going to combine the analysis for different study design)”*
  - Thanks for the comment! To dissipate ambiguities, we have added an explanation of how and under which case estimates are going to be extracted from interventional studies. (Line 125-128)

**Reviewer #3:**

- Comment 1: *“The authors are planning to perform a systematic review and meta-analysis examining the prognostic value of early high-sensitivity troponin in predicting short-term mortality in sepsis based on a detailed, comprehensive, and clear protocol that has been registered in PROSPERO (CRD42024468883). I only have 2 minor comments on the Methods section: Selection criteria. Page 7, Table 3. One of the inclusion criteria only limits published articles in English language. To improve the inclusion of more potential articles, the authors may consider not to have any language restrictions.”*
  - Response: We agree with the reviewer; the respective modifications can be found above as the very same concern was raised by reviewer 1. (Line 94, Table 3).
- Comment 2: “Page 9. Table 3.

The authors plan to include published studies, conferences, and abstracts. To ensure that the quality of included studies are of higher standards, only published studies in the form of full article should be included in the systematic review and meta-analysis. Please consider excluding abstracts presented in conferences”

- Response: Thanks to the reviewer for the constructive comment. The authors have thoroughly debated both the reviewer´s and the manuscript´s positions, and we have finally decided to follow the original inclusive criteria. Although it is true that restricting the participation to published papers only guarantees, as the reviewer notes, a high-quality standard, we have come to the conclusion that limiting the future review to these publications might increase the risk of publication bias since it has been noted that dissertations and thesis present on average more conservative estimates than the published literature (Please see reference 1-2). Our decision aligns with the Cochrane Group recommendations for diagnostic test accuracy studies meta-analysis, which is the recommended handbook for prognosis studies meta-analysis at this moment (Reference 3).

**Reviewer #4**

- Comment 1: *“My only remark relates to the sentence at line 156 : "Authors will be contacted to request critical missing or not reported data regarding associations of interest. In case of no response, the article will be excluded from this study." I wonder if the authors could find a way to include at least some data from these incomplete articles, if there were any, instead of excluding them a priori.”*
  - Response: We thank the reviewer for the comment. In response, we have clarified the nuances of the stated paragraph, whereby now it is noted what kind of missing data is regarded as critical (ie. PICOTS assessment data) (Line 163). In our opinion, the exclusion of these articles is appropriate since the pertinence of their findings to the very specific setting –this doesn't pretend to be a *general* appraisal of prognostic troponin – being evaluated is ill-defined and might introduce substantial bias. We have acknowledged the possibility of missing pertinent papers due to a lack of critical data reporting as a limitation of the review in the *Discussion* segment, as mentioned above. (Line 268 - 275).

We hope all these changes and the improvement of the quality of the new manuscript version contribute to response the reviewer’s comments in a good manner.

If you have any other comments, please do not hesitate to let us know, and we will address them promptly.

Best Regards,

**Abraham Gajardo Cortez, MD-PhD; Santiago Ferriere-Steinert, BSc; Sebastian Heskia Araya, BSc; Thomas Kouyumdijan, BSc; Joaquín Valenzuela, BSc; José Tomás Rojas, BSc**

**Hospital Clínico Universidad de Chile**

**Faculty of Medicine**

**University of Chile**

References

1)Zimpel T, Windeler J. Veröffentlichungen von Dissertationen zu unkonventionellen medizinischen Therapie- und Diagnoseverfahren - ein Beitrag zum <<publication bias>> [Publications of dissertations on unconventional medical therapy and diagnosis procedures--a contribution to "publication bias"]. Forsch Komplementarmed Klass Naturheilkd. 2000 Apr;7(2):71-4. German. doi: 10.1159/000021324. PMID: 10899742.

2)Mary Lee Smith,

Publication bias and meta-analysis,

Evaluation in Education,

Volume 4,

1980,

Pages 22-24,

ISSN 0191-765X,

https://doi.org/10.1016/0191-765X(80)90004-X.

(https://www.sciencedirect.com/science/article/pii/0191765X8090004X)

3) Deeks JJ, Bossuyt PM. Chapter 2: Evaluating medical tests.In: Deeks JJ, Bossuyt PM, Leeflang MM, Takwoingi Y editor(s).Cochrane Handbook for Systematic Reviews of DiagnosticTest Accuracy Version 2.0 (updated July 2023). Cochrane,2023. Available from https://training.cochrane.org/handbook-diagnostic-test-accuracy/current.
